# Hemorrhagic hypopyon as presenting feature of intravascular lymphoma, a case report

**DOI:** 10.1186/s12886-017-0591-3

**Published:** 2017-10-25

**Authors:** Andrew Winegarner, Noriyasu Hashida, Shizuka Koh, Kohji Nishida

**Affiliations:** Department of Ophthalmology, Osaka University Medical School, E7, 2-2 Yamadaoka, Suita, Osaka, 565-0871 Japan

**Keywords:** Herpes simplex uveitis, Intravascular lymphoma, Ophthalmic manifestations, Vessel permeability, Hemorrhagic hypopyon, Case report

## Abstract

**Background:**

Herpes uveitis has been previously reported to present with hyphema, but hemorrhagic hypopyon is rarely reported as a herpetic uveitis manifestation. We report a case of herpes simplex virus (HSV) presenting with hemorrhagic hypopyon, and speculate on the underlying pathophysiology with relation to an intravascular lymphoma which was subsequently diagnosed as a result.

**Case presentation:**

We present a case wherein a 62-year-old Japanese rheumatoid arthritis woman, with HSV uveitis, presented with hemorrhagic hypopyon in the anterior chamber and a fever with photophobia. Patient was treated with antiviral drugs which improved the hyphema and corneal lesions, but lesions recurred 3 months later. This rare presentation of HSV induced uveitis, and its subsequent recurrence, aroused suspicion of an additional hypopyon-inducing pathology. On account of previous history of lung opacities and elevated LDH, intravascular lymphoma was eventually diagnosed via lung biopsy. She was treated for the lymphoma which also completely resolved all ocular symptoms without any recurrence as of 1.5 years later.

**Conclusion:**

The exceedingly rare presentation of hemorrhagic hypopyon may have been enabled by an interaction of the HSV with the intravascular lymphoma. HSV involvement was indicated by the dendritic lesions, IgG assay, and response to anti-viral drugs. The ocular involvement of the intravascular lymphoma seems to be indicated by virtue of the anti-tumor drugs completely resolving all ocular symptoms.

## Background

Herpes Simplex Virus (HSV) will usually be asymptomatic during its initial infection, and remains latent thereafter. Herpetic uveitis can have several manifestations such as keratitis, corneal edema, and iris atrophy. Hyphema has previously been reported to manifest in herpes simplex virus with cutaneous lesions [1] and herpes zoster uveitis [2], but hemorrhagic hypopyon remains unreported. The rarity of even non-hemorrhagic hypopyon HSV presentation is demonstrated by current literature cautioning against mistaking hypopyon as a sign of HSV disease, instead suggesting one should seek alternate explanations for the hypopyon.

Intravascular lymphomas are a rare subtype of diffuse large B-cell lymphomas and sometimes T-cell lymphomas, first reported by Pfleger in 1959 [3]. These lymphomas do not generally present with lymphoma cells in the lymph nodes, and they often spare the surrounding tissue in which they are located. Generally restricted to the intraluminal space, these lymphomas are difficult to detect early in their progression. Consequently, proper diagnosis is often delayed and mortality rates are high [3].

In the current study, we report a case of intravascular lymphoma which is diagnosed early via biopsy after herpetic uveitis presentation aroused suspicion of an ulterior pathophysiology. The patient had hemorrhagic hypopyon presenting in the anterior chamber. The rarity of this clinical picture gave reason to search for a more satisfactory diagnosis than simply HSV uveitis. A lung computerized tomography (CT) scan and subsequent biopsy revealed an intravascular B cell lymphoma. This unique clinical presentation potentially elucidates a mechanism by which intravascular lymphoma pathophysiology may interplay with other pathologies, such as HSV uveitis.

## Case presentation

A 62-year-old Japanese woman was referred for bilateral dry eyes, with Schirmer’s test results of 0 mm for both eyes. Four years previously, she had been diagnosed with primary rheumatoid arthritis (RA) and secondary Sjogren’s syndrome. She was being treated with 6 mg of methotrexate. CT scans appeared normal when she was first diagnosed with RA. Just prior to her dry eye referral, her previous hospital found C-reactive protein (CRP) and lactate dehydrogenase (LDH) levels to be significantly increased (CRP of 11.83 and LDH of 952), leading to a suspicion of an underlying malignancy. A CT scan was done, and some lung opacities were found, but biopsies of bone marrow, pleural fluid and cerebrospinal fluid (CSF) all revealed no neoplastic activity.

**Fig. 1:**
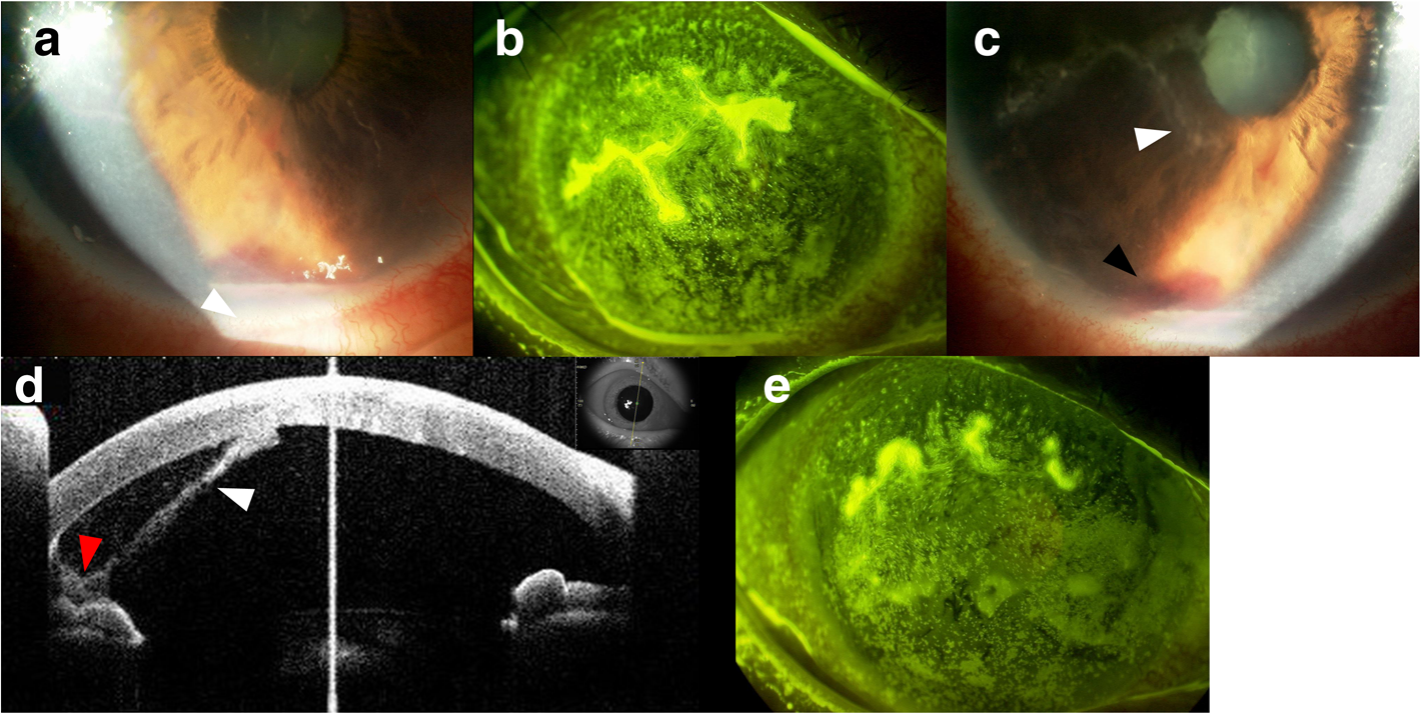
**a** Hemorrhagic hypopyon can be seen resting in the anterior chamber. **b**The herpetic corneal dendrites and opacity can be seen on the corneal surface via fluorescein staining. **c** A magnified image reveals the hemorrhagic hypopyon (bottom arrow) and fibrin deposits (top arrow) more clearly. **d** Anterior segment OCT demonstrates the accumulating hypopyon (red arrow) and fibrin deposition (white arrow). **e** A fluorescein staining of the cornea demonstrating herpetic dendrites and corneal opacity at the time of recurrence 3 months after initial admission

While being treated for dry eye at our hospital, emergent hyperemia presented in the right eye coupled with fever and photophobia, for which she was admitted to the hospital. In the inpatient ward, hemorrhagic hypopyon that showed niveau-like hypopyon with hemorrhage as reported in previous case [4] was seen on slit lamp(Fig. 1a). A distinct herpetic corneal lesion (Fig. 1b) were noted via fluorescein staining. Keratic precipitates were noted, but no synechiae. HSV was specifically identified in the right eye via Checkmated Herpes-eye®, (Wakamoto Co., Ltd., Tokyo Japan). Corneal smear was done to rule out bacteria as a potential cause. Right eye visual acuity was found to be 20/500, while left eye visual acuity remained 20/20. Right eye intraocular pressure (IOP) was 16 mmHg and left eye IOP was 18 mmHg. The anterior segment of the right eye presented with hyperemia, dendritic keratitis, and intracameral fibrin deposits (Fig. 1c). Posterior segment appeared normal. Anterior segment optical coherence tomography (AS-OCT) further illustrate the fibrin deposits and hemorrhagic hypopyon (Fig. 1d). Body temperature was elevated to 38.5 °C, with a white blood cell count (WBC) of 10.4 × 10^3^/μL (with neutrophils being 64.3%, lymphocytes 16.5%, monocytes 16.8%, eosinophils 1.9%, and basophils 0.5%) Treatment with topical acyclovir ophthalmic ointment (5 times daily), topical levofloxacin (5 times daily) and oral valacyclovir (3 times daily). Two days later, the dendritic keratitis began to regress.

By day 5 of treatment, the dendritic keratitis, fibrin, hypopyon had regressed, with only superficial, punctate keratitis and Descemet membrane folds remaining. WBC normalized, CRP dropped to 9.72 and body temperature dropped to 36 °C. The right eye’s visual acuity recovered to 20/200, which was comparable to baseline on account of a cataract. Three weeks after hospitalization, symptoms had completely regressed; slit-lamp findings revealed no findings indicative of herpetic keratouveitis or recurrent hypopyon.

**Fig. 2:**
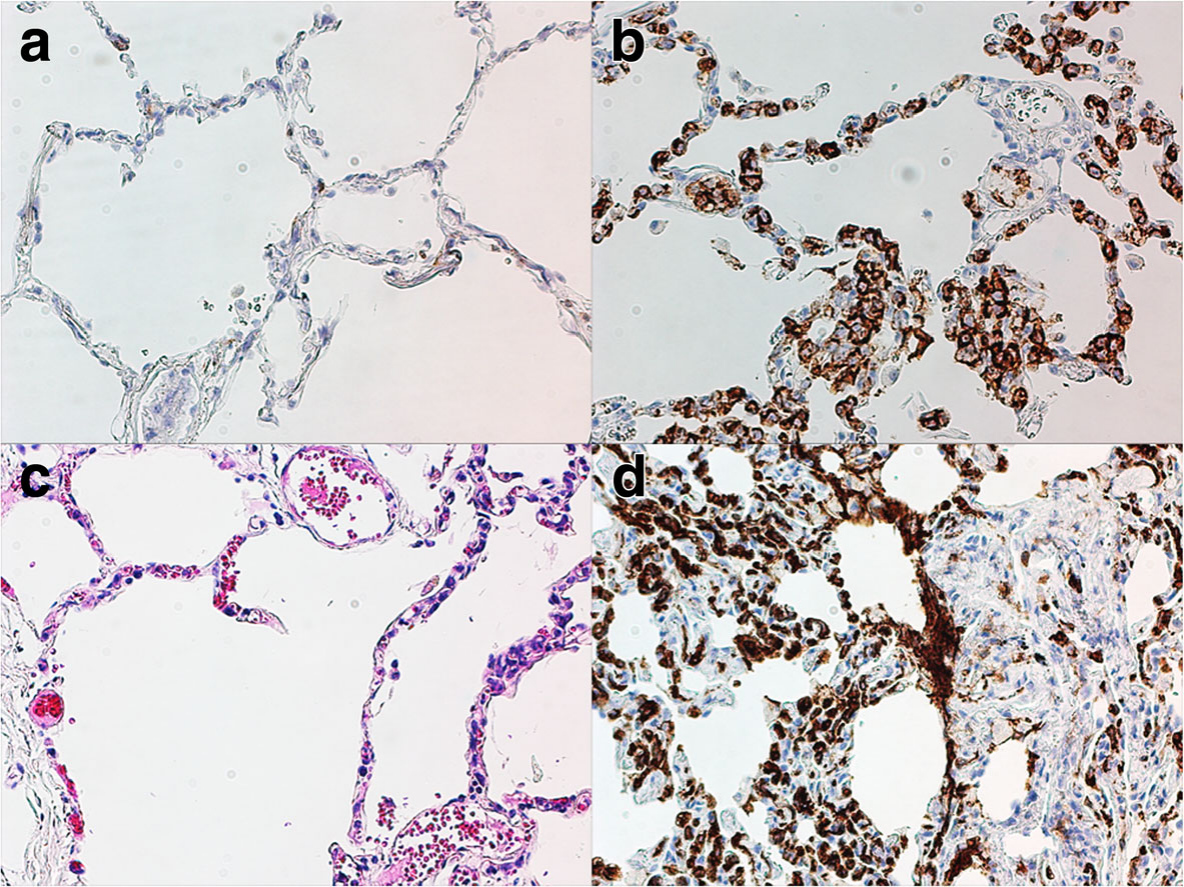
**a** A CD3 stain of the lung biopsy reveals no T cell involvement. **b** A CD20 stain reveals heavy staining within the lumen and alveoli, indicating a B cell lymphoma. **c** H&E stain demonstrates lymphocytic infiltration along the alveoli and in the lumen. **d** CD79 stain further confirms heavy involvement of B cells indicating the intravascular lymphoma is of B cell origin

**Fig. 3:**
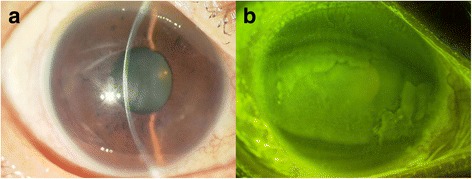
**a** Slit lamp photograph from 1.5 years post treatment reveals no hypopyon. **b** Corneal fluorescein staining demonstrated no significant opacification or herpetic lesion

Nearly 3 months later, a fever and corneal opacities reoccurred. Again, a characteristic herpetic dendritic lesion, superficial punctate keratitis, and intracameral fibrin deposits were noted (Fig. 1e). WBC count remained normal, diminishing suspicion of RA flare-up. Due to the previously elevated LDH levels and previous rare viral presentation, malignancy was again suspected. A positon-emission tomography-computed tomography (PET-CT) showed significant diffuse lung opacities whereafter a lung biopsy was performed to confirm an intravascular B cell lymphoma (Fig. 2a-d). Rituximab, cyclophosphamide, doxorubicin, vincristine, and prednisolone (R-CHOP) regimen was begun immediately, and the corneal lesions and the lymphoma both went into complete remission. Over a year and a half later, there is still complete remission of the cancer and no corneal lesions or ocular symptoms (Fig. 3a-b).

## Discussions

Intravascular lymphomas have been reported to be associated with various complications in the eye (vitreous opacities, vein occlusions etc.). More generally, lymphomas have been documented as an underlying cause of both pseudo-hypopyon, hypopyon and hyphema in previous reports [5–9]. This coupled with the previously elevated LDH was the main reason we suspected something akin to a lymphoma might be complicating the atypical presentation of what was suspected to be an HSV infection. The HSV itself seems to have played a role as well, as evident by the antiviral drugs causing the hemorrhagic hypopyon to regress, in addition to the positive HSV specific immunochromatography and diagnostic corneal lesions. Not entirely different from Dr. Okuniki’s case report concerning herpes zoster uveitis and its presentation of hyphema [2]. It is likely that two types of conditions such as HSV uveitis and intravascular lymphoma existed simultaneously. We performed systemic survey such as whole body CT and blood test including CRP to check and rule out endogenous infection. This patient systemically proved to have HSV in serum, also on ocular surface, and had rare association of hemorrhagic hypopyon. According the previous paper, infectious uveitis with HSV showed severe hyphema not hemorrhagic hypopyon; therefore, we suspected accumulated materials in the anterior chamber were hyphema plus tumor cell and/or inflammatory cells. Moreover, upon the beginning R-CHOP therapy, the ocular symptoms completely resolved with no recurrence, supporting out suspicion that the intravascular lymphoma did in fact play a role concurrently with the HSV in the patient’s ocular clinical presentation.

It is likely that the association of the herpetic uveitis with an intraocular lymphoma might cause rare clinical manifestation of the mixture of hyphema and hypopyon. Because, the patient was immunocompromised host at the emergence of intravascular lymphoma and these conditions allow the HSV to show corneal lesions and hyphema plus hypopyon in anterior chamber. Concerning the association of intravascular lymphoma, it is possible that the blood-aqueous barrier may have been rendered permeable by the intravascular lymphoma, allowing the inflammatory cells and red blood cells to leak into the anterior chamber, causing the hemorrhagic hypopyon. Intravascular lymphomas have been reported to cause anasarca as a symptom and even initial presentation. The speculated mechanism is an increase in vascular permeability due to direct damage of endothelial cells and blockage of the vessel by the lymphoid cells [10]. Additionally, vessel dilation has been reportedly seen with intravascular lymphomas directly [11]. The proposed means by which the hemorrhagic hypopyon arose would be similar, wherein dilation, and damage of vessels would cause the inflammatory cells to possibly leak into the anterior chamber.

There have been several reports linking RA and methotrexate treatment to increased incidence of lymphomas, perhaps offering a clue as to the initial predisposing factors for the intravascular lymphoma [12]. Additionally, RA and methotrexate have recently been explicitly linked to a case of intravascular lymphoma [13]. Immunosuppression from the methotrexate and the lymphoma may have allowed the HSV to exit latency. The intravascular lymphoma possibly caused increased vessel permeability, allowing the HSV inflammatory response to enter the anterior chamber causing the hemorrhagic hypopyon. It is likely that herpetic uveitis could be the only cause of hemorrhagic hypopyon and intraocular lymphoma could be a possible uncertain secondary cause of hemorrhagic hypopyon, however; we think the patient had coexisting clinical conditions such as HSV-1 infection and intraocular lymphoma, simultaneously. Because, at the time of first manifestation of ocular lesion, we treated only with anti-viral drugs but we could not achieve complete remission, though at the time of second recurrence we evaluated systemic conditions and treated systemically with anti-tumor drugs, not using anti-viral drugs and we could achieved complete remission. These facts suggest neoplastic conditions, at least, partially related to the manifestation of ocular lesions. We could not know detail mechanism of hypopyon formation; however, it is likely that immunosuppressed conditions in the patient allow to coexist two clinical manifestation such as hyphema case by HSV infections and hypopyon caused by intravascular lymphoma.

## Conclusion

In conclusion, the peculiar presentation of anterior chamber hemorrhagic hypopyon coupled with symptoms of herpetic uveitis may have arisen from an interaction between the intravascular lymphoma and the HSV. Ultimately, we were able to detect, diagnose, and treat the intravascular lymphoma on account of the peculiar ocular presentation. Potentially offering insight into the pathophysiology of intravascular lymphomas, and a precedent for parsing out causes and interactions in rare and unprecedented clinical presentations.

## Abbreviations

AS-OCT: Anterior segment optical coherence tomography
CRP: C-reactive protein
CSF: Cerebrospinal fluid
CT: Computerized tomography
HSV: Herpes simplex virus
IOP: Intraocular pressure
LDH: Lactate dehydrogenase
RA: Rheumatoid arthritis
RCHOP: Rituximab, cyclophosphamide, doxorubicin, vincristine, prednisone
WBC: White blood cell count
